# The Role of Urinary Microbiome Analysis in the Diagnostic Approach and Management of Urinary Incontinence: A Systematic Review

**DOI:** 10.3390/life15020309

**Published:** 2025-02-17

**Authors:** Pouyan Shaker, Zohreh Roshani, Ehsan Timajchi, Zahra Sharifi, Sarah Nikzadfar Goli, Behrooz Broumand, Mohammad Ali Shafiee

**Affiliations:** 1 College of Medicine, Kansas City University, Kansas City, MO 64106, USA; pouyan.shaker@kansascity.edu; 2 University Health Network (UHN), Toronto, ON M5T 2S8, Canada; zohreh.roshani3@gmail.com (Z.R.); zahrasharifi.uni@hotmail.com (Z.S.); 3 Department of Human Biology, University of Toronto, Toronto, ON M5S 3J6, Canada; ehsan.timajchi@mail.utoronto.ca; 4 School of Arts, Sciences and Education, D’Youville University, Buffalo, NY 14201, USA; nikzas18@dyc.edu; 5 Nephrology Unit of Pars Hospital, Tehran 1415944911, Iran; v4broumand@yahoo.com; 6 Division of General Internal Medicine, Department of Medicine, University of Toronto, Toronto, ON M5S 1A8, Canada

**Keywords:** urinary incontinence, microbiome, urgency urinary incontinence, overactive bladder, 16S rRNA sequencing, personalized therapy

## Abstract

Urinary incontinence (UI) is a significant global health issue that impacts mainly middle-aged women, severely affecting their quality of life. Emerging research highlights the urinary microbiome’s complex role in the etiology and management of UI, with microbial dysbiosis potentially influencing symptom severity and treatment outcomes. This systematic review aimed to evaluate the current evidence on the urinary microbiome’s role in diagnosing and managing UI, focusing on variations in microbial composition across UI subtypes. We identified 21 studies, mostly employing 16S rRNA sequencing to characterize urinary microbiota and their associations with various UI subtypes, including urgency urinary incontinence (UUI), overactive bladder (OAB), and stress urinary incontinence (SUI). The findings revealed distinct microbial patterns, such as reduced Lactobacillus levels and increased Gardnerella prevalence, particularly in UUI. Altered microbiome profiles correlated with symptom severity, with reduced Lactobacilli suggesting a protective role in maintaining urinary health. Specific microbial species, including Actinotignum schaalii and Aerococcus urinae, emerged as potential biomarkers for UI diagnosis. Despite promising findings, limitations such as small sample sizes, variability in microbiome profiling methods, and insufficient causal evidence underscore the need for further research.

## 1. Introduction

Urinary incontinence (UI) is a prevalent and debilitating lower urinary tract condition that significantly impacts the quality of life of those affected. The prevalence of UI is approximately 45%, with prevalence increasing with age from 28% in women aged 30 to 39 years to 55% in women aged 80 to 90 years [1]. It is characterized by the involuntary loss of urine and encompasses symptoms such as frequent urination, urgency, and nocturia. Traditionally, the urinary tract was considered a sterile environment; however, recent studies have revealed a complex and dynamic microbial community within the urinary system [2]. This evolving understanding suggests that alterations in the urinary microbiome may contribute to the pathogenesis of various UI subtypes, including urgency urinary incontinence (UUI), overactive bladder (OAB), stress urinary incontinence (SUI), and mixed urinary incontinence (MUI), which are distinguished by their clinical characteristics [3]. SUI involves urine leakage triggered by physical exertion, such as coughing, sneezing, or exercising. UUI is defined by a sudden and intense urge to urinate, often leading to involuntary leakage before reaching the restroom. OAB, closely related to UUI, is characterized by urgency with or without leakage, often accompanied by frequency and nocturia [4].

MUI combines symptoms of SUI and UUI, presenting challenges for diagnosis and treatment due to its multifactorial nature. Other less common subtypes include functional incontinence, where physical or cognitive limitations prevent timely access to the restroom, and overflow incontinence, caused by incomplete bladder emptying [5].

Although age, menopause, body mass index (BMI), and parity are well-established risk factors for UI, the underlying pathophysiology remains poorly understood [6]. Emerging research suggests that the urinary microbiome may play a key role in these mechanisms. The microbiome’s interaction with the immune system is thought to influence symptom severity, providing a potential new method for diagnosis and treatment [7].

Advances in microbial detection techniques, such as 16S rRNA gene sequencing and expanded quantitative urine culture (EQUC), have challenged the traditional concept of sterile urine in healthy individuals, revealing a diverse microbial ecosystem within the urinary tract [8]. These technologies allow for the identification of bacteria and other microorganisms, including those that are not detectable by standard culture methods.

Maintaining a delicate microbial balance within the urinary tract is critical for urinary health. Disruptions to this balance, or dysbiosis, have been associated with various urinary conditions, including UUI and OAB [9]. The urinary microbiome includes diverse bacterial genera such as Lactobacillus, Corynebacterium, Streptococcus, Actinomyces, Staphylococcus, Gardnerella, and Bifidobacterium. A greater abundance of Lactobacillus is linked to a healthier urinary environment, while an overrepresentation of bacteria like Gardnerella and Prevotella is associated with UUI and other urinary symptoms [10]. This growing body of evidence underscores the importance of microbial homeostasis in maintaining urinary tract health and highlights dysbiosis as a potential contributor to UI pathogenesis.

The role of the urinary microbiome in diagnosing and managing UI is a growing area of research. Understanding how microbiome composition varies across UI subtypes, such as UUI, OAB, and SUI, could pave the way for innovative diagnostic tools and personalized treatment strategies. By identifying microbiome-based biomarkers, researchers hope to improve early detection and develop targeted therapies that restore microbial balance without relying on antibiotics [6].

This systematic review aimed to synthesize the existing evidence on the role of the urinary microbiome in diagnosing and managing urinary incontinence. Specifically, it explores microbiome differences across UI subtypes and their influence on management.

## 2. Materials and Methods

This systematic review adheres to the guidance of the Cochrane Collaboration and follows the Preferred Reporting Items for Systematic Review and Meta-analyses (PRISMA) statement. The research protocol was registered in PROSPERO with the assigned registration number CRD42025633823. Embase, Medline, PubMed, PsychInfo, and CINAHL were searched using their respective database search engines up to September 2024.

A comprehensive search was conducted to identify studies examining the relationship between urinary incontinence (UI) and the urinary microbiome. The search strategy utilized Medical Subject Headings (MeSH) terms and keywords related to UI, bladder dysfunction, and the urinary microbiome. Terms such as urine incontinence, incontinence, overactive bladder, bladder disease, urinary dysfunction, and bladder dysfunction were used to capture all relevant studies on the relationship between the bladder microbiome and UI. To identify research focusing on the microbiome, terms including microbiome, biome, bacterial microbiome, bladder microbiota, and urinary tract microbiota were employed.

The search combined terms related to UI and the microbiome using the Boolean operator “AND.” Results were refined by applying filters to include only studies conducted on humans and published in English. Additionally, the inclusion criteria specified that the study population must consist of women aged 18 years and older. Manual searches were performed through the reference lists of included articles and relevant citations retrieved from the primary search results to complete the electronic search. Studies were included if they provided data on the relationship between bladder microbiota and UI. Exclusion criteria encompassed studies involving animals or children and research conducted primarily in laboratory settings, as well as non-English articles, reviews, editorials, surveys, poster presentations, and abstracts from major journals.

Two researchers independently screened the titles and abstracts of all retrieved studies to identify those eligible for inclusion for full-text screening and further data extraction. In cases of uncertainties or disagreements during this initial screening, a senior reviewer was consulted to resolve the issue and reach a final decision. Following this, data from each included study were systematically extracted and recorded in a predesigned data extraction spreadsheet. This spreadsheet captured key details, including the primary author, publication year, study design, age and presence of urinary incontinence, microbiome analysis techniques, and any reported associations between the bladder microbiome and urinary health outcomes.

The methodological quality of the included studies was assessed using the modified Newcastle–Ottawa Scale (NOS). Each study was assigned a score ranging from 0 to 9, with higher scores indicating better methodological quality. The assessment focused on criteria such as sample representativeness, group comparability, and outcome measurement.

## 3. Results

### 3.1. Study Characteristics and Demographics

**Figure 1:**
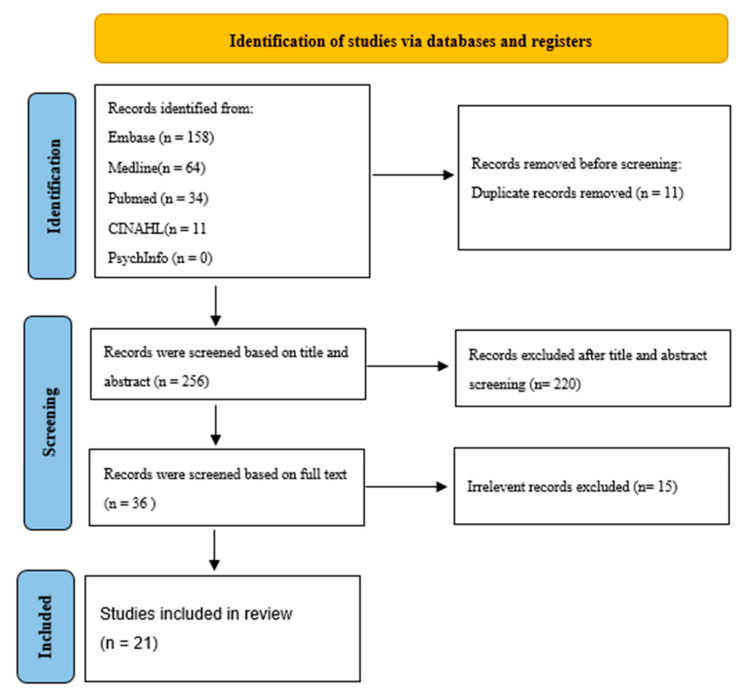
PRISMA flow diagram [11] study selection process.

Using a comprehensive search strategy, a total of 256 studies were identified after duplicates were removed. Following the screening of titles and abstracts, excluding duplicates and non-human studies, 36 articles were selected for full-text review. Figure 1 shows the Preferred Reporting Items for Systematic Reviews and Meta-Analyses (PRISMA) flowchart, outlining the study selection process. Of the selected articles, 15 papers were excluded due to irrelevance or missing data, resulting in 21 studies being included in the final review.

The final selection comprised studies published from 1947 to 30 December 2024, with participant sample sizes ranging from 9 to 1004, totaling 3125 participants across all studies. However, the earliest study meeting our inclusion criteria and included in the final analysis was published in 2015. Table 1 summarizes the characteristics of the included studies. The majority were cross-sectional (seven studies), followed by case–control studies (four studies); there were also three prospective cohort studies and three clinical trials, including a quasi-experimental trial. Participants primarily consisted of women aged 18 and older with urinary incontinence, with mean ages ranging from 37.1 years to 75 years.

### 3.2. Microbiome Analysis and Incontinence Outcomes

Microbiome analysis methods varied across studies, most utilizing 16S rRNA gene sequencing to characterize bacterial communities. Diversity in microbiome composition was reported, with significant variations observed between participants with and without urinary incontinence. The studies reported a consistent association between the severity of the urinary microbiome and urinary incontinence. Specifically, several studies found that lower levels of Lactobacilli were correlated with increased symptom severity.

### 3.3. Impact of Microbiome on Incontinence Types

This review highlighted differences in microbiome profiles among various types of incontinence. One study showed that women with urinary incontinence demonstrated higher bacterial abundance and richness, with specific species such as Actinotignum schaalii and Aerococcus urinae more prevalent in UI cohorts. Microbial diversity, however, was not significantly different between UI patients and controls, suggesting that bacterial richness rather than diversity may be linked to symptom severity. For example, studies focusing on OAB and UUI indicated distinct bacterial compositions compared to controls. Common findings included a dominance of Lactobacillus spp. in most samples, with some studies identifying other bacterial species as potential contributors to symptom manifestation.

### 3.4. Methodological Quality

The methodological quality of the included scores ranged from 4 to 9. Most studies met the sample representativeness, comparability, and outcome assessment criteria. However, a few studies reported limitations, including potential biases in microbiome sample collection methods and small sample sizes.

## 4. Discussion

UI is a significant public health problem affecting millions of people worldwide. This study systematically reviewed the growing body of literature investigating the role of the urinary microbiome in the etiology and treatment of UI among adult women. We reviewed 22 studies that used 16S rRNA gene sequencing to comprehensively assess how microbiome profiling contributes to understanding UI pathophysiology. These studies have characterized the complex bacterial communities within the urinary tract, revealing complicated relationships between the composition of urinary microbiota and UI symptoms and extending the way to new diagnostic and therapeutic approaches.

One study showed significant differences in the urinary microbiome of patients with OAB with detrusor overactivity compared to OAB patients without detrusor overactivity and matched controls. Specifically, studies indicated that OAB patients with detrusor overactivity tend to have a less diverse microbiome and a higher proportion of Lactobacillus, particularly *Lactobacillus iners* [14]. These findings align with the broader understanding of microbial dysbiosis in OAB and suggest that the urinary microbiome may be involved in the pathogenesis of a specific phenotype of OAB.

The findings from the other included studies indicated an inverse correlation between *Lactobacillus* and the severity of urinary incontinence, suggesting a protective role of *Lactobacilli* in sustaining a healthy urinary tract [12,21,32]. *Lactobacillus* species, including *L. iners*, are known to help maintain a balanced microbial environment by preventing UTIs. The mechanism outcompetes pathogenic bacteria, such as *Escherichia coli*, for resources and adhesion sites on the urinary tract lining [31]. The underpresentation of *Lactobacillus* in OAB patients with detrusor overactivity may indicate an altered microbial balance that could contribute to the onset or exacerbation of OAB symptoms [31]. Therefore, understanding how microbial dysbiosis influences the severity of OAB symptoms may lead to new therapeutic strategies targeting the urinary microbiome. Additionally, the results from different studies revealed that bacterial richness might be more important than diversity in determining the severity of UI [20,32]. *Actinotignum schaalii* and *Aerococcus urinae* were two specific bacterial species that showed higher prevalence in patients with UI, indicating their potential as microbial biomarkers for diagnosing specific UI subtypes [23]. This finding could promote the development of more personalized diagnostic and therapeutic strategies for UI management.

This review showed that different treatment techniques are necessary for incontinence categories such as OAB and UU since they are associated with variations in microbiota profiles. These groups’ overrepresentation of specific bacterial species highlights the value of microbiome studies in creating and overseeing patient care programs. This fact introduces the prospect of employing treatments for individual patients. In other words, altering the urine microbiota would be a novel therapeutic approach, especially for individuals who do not react to conventional medications. The correlations between the urine microbiome and the severity suggest the opportunity for microbiome-targeted therapies. Interventions aimed at restoring healthy *Lactobacillus* levels or modulating specific pathogenic species could offer novel treatment avenues for UI. Therefore, microbiome profiling could be useful for determining who is more susceptible to UI or for developing individualized treatment plans.

It is important to note that current treatments for OAB remain limited to medications and behavioral interventions, particularly for patients with severe symptoms [19]. However, microbiome-targeted therapies could offer a novel avenue for those who do not respond well to traditional treatments. Given the variations in UI subtypes, microbiome profiling may help tailor treatment, including in patients who do not respond to conventional therapies.

The studies included in this review employed various methods for microbiome analysis, predominantly utilizing 16S rRNA gene sequencing. This approach provides a comprehensive characterization of bacterial communities and allows for the identification of specific taxa associated with UI. However, differences in sample collection methods, such as the use of catheterized versus midstream urine samples, may introduce variability in microbiome profiles and impact the comparability of results. On the other hand, despite the promising findings, several controversies and gaps remain in the current understanding of the urinary microbiome’s role in UI. For example, the complexity of polymicrobial interactions poses challenges for establishing definitive causal relationships between specific microbiome profiles and urinary disorders. Additionally, the influence of host factors, such as hormonal changes and age, further complicates these interactions, suggesting a need for continued research to unravel these dynamics [6].

When considering patients with positive urine cultures or analyses suggesting infection, it is important to distinguish whether the urinary microbiome changes are due to infection or an underlying cause of UI [33]. Pathogens like *E. coli* confound microbiome analysis, and their role in driving or merely coexisting with UI symptoms needs further clarification. Future research should aim to separate the effects of infection from dysbiosis to better understand the microbiome’s role in UI.

The modified NOS ranged from moderate to high quality for the included studies. Most studies adequately addressed sample representativeness, comparability, and outcome assessment. However, common limitations in included studies were small sample sizes and potential biases in microbiome sample collection, which may affect the generalizability of findings. Additionally, the variability in analytical techniques emphasizes the importance of adopting uniform methods to improve the reliability of results. Therefore, future studies should standardize sample collection protocols and include larger, more diverse populations to enhance the validity and reliability of results.

## 5. Conclusions

This review highlights the role of the urinary microbiome in UI. We found that Lactobacillus iners is more prevalent in OAB with detrusor overactivity, while reduced Lactobacillus levels correlate with greater UI severity. The presence of Actinotignum schaalii and Aerococcus urinae suggests potential microbial biomarkers for diagnosis. Considering the variation in microbiota compositions observed in different UI subtypes, microbiome profiling can optimize treatment selection, specifically for patients who do not respond to conventional therapies. However, inconsistencies in sample collection methods and analytical approaches currently hinder the reliability and applicability of existing findings. Future studies should standardize methodologies and include diverse populations to validate these approaches and enhance clinical applications.

## Figures and Tables

**Table 1:** Characteristics of studies included in the systematic review.

Authors and Year	Study Design	Population/ Participants and Sample Size	Statistical Analysis	Incontinence Severity Measures	Sample Microbiome Analysis	Mean Age (SD)	Method of Microbiome Analysis	**Results**	**Conclusion**	**NOS**
Carnes, 2024 [12]	Secondary analysis cross-sectional study	Women with urinary incontinence (N = 126)	Linear regression models adjusted for age and BMI. Links between microbiome community types and incontinence severity.	Bladder diaries and UDI scores	Both urine and vaginal	53 years (10.7)	Sequencing of 16S rRNA V4 to V6	Fewer Lactobacilli were linked to higher total leaks (2.67 daily total leaks; 95% CI, 0.76–4.59; *p* = 0.007) and urgency leaks (1.75 daily leaks; 95% CI, 0.24–3.27; *p* = 0.02). No notable links were found for stress urinary incontinence episodes or UDI scores.	Urinary microbiome community types, particularly those with fewer Lactobacilli, were related to increased urinary incontinence severity	7
Gabriel, 2023 [13]	Prospective pilot study	Women UUI who were prescribed treatment with anticholinergics (N = 20)	Baseline and outcomes were summarized as median (IQR) for continuous data and percentages for categorical data. Non-normality was confirmed, and statistical differences were assessed using Wilcoxon, Mann–Whitney U, Fisher’s exact, and chi-square tests.	UDI-6 questionnaire, 2-day bladder diary	Catheterized urine samples	Median = 64 years (IQR: 52.5–73.25)	16S rRNA gene sequencing (V4 hypervariable region), microbiologic urine culture, and cytokine analysis	Lactobacillus was the most common organism at baseline and follow-up. In total, 42% of patients responded to anticholinergics based on UDI-6 scores. No significant changes in urine cytokine levels or correlation between baseline microbiome and treatment response. Lactobacillus abundance varied post-treatment.	Urine microbiome and inflammatory markers showed no significant correlation with treatment response to anticholinergics for UUI, suggesting that other factors may influence treatment outcomes.	
Javan Balegh Marand, 2023 [14]	Case–control study	Female patients with OAB with and without detrusor overactivity (N = 33): 21 e patients with OAB, 12 with OAB DO+ and 9 with OAB DO-; 12 healthy, age- and sex-matched controls	Bacterial proportions were calculated. Genera considered ‘present’ if >0.001%. The SDI assessed abundance; normality was tested using Kolmogorov–Smirnov and Shapiro–Wilk tests. ANOVA and Kruskal–Wallis tests with pairwise comparisons assessed group differences.	No incontinence severity measures	Midstream urine	OAB DO+ group: 57 years (range 31–72); OAB DO- group: 56 years (range 24–74); Control group: 53 years (range 38–61)	16S rRNA V1–V2 Region Amplification	Higher Lactobacillus, especially Lactobacillus iners, was linked to reduced microbiome diversity in detrusor overactivity (DO+) patients. OAB DO+ patients had a significantly lower median SDI (1.75) compared to OAB DO- (3.34; *p* = 0.006) and controls (3.39; *p* = 0.003)	Urinary microbiome community types, particularly those dominated by Lactobacillus iners and characterized by reduced diversity, are associated with detrusor overactivity and may contribute to the severity of overactive bladder symptoms.	6.5
Joyce, 2022 [15]	Cross-sectional study	Women with various urinary tract symptoms (N = 1004). Cases (N = 658): UTI (N = 304), UUI (N = 255), SUI (N = 50), IC/PBS (N = 49); Controls (N = 346)	Age-adjusted models assessed microbial abundance, prevalence, and diversity, with significant cohort differences determined using Sidak-corrected *p*-values (<0.05). Alpha diversity was regressed on age and cohort, and Spearman’s rho estimated age and BMI correlations. Genus abundance and prevalence were analyzed using negative binomial and logistic regression.	Not reported	Catheterized urine samples	All Patients: 59 years (16) UUI: 65 years (12) SUI: 54 years (14)	Expanded Quantitative Urine Culture (EQUC) followed by Matrix-Assisted Laser Desorption Ionization–Time-of-Flight Mass Spectrometry (MALDI-TOF MS) for bacterial identification.	UUI showed higher prevalence of various genera, including Enterococcus, Corynebacterium, Lactobacillus, Streptococcus, Actinomyces, and Gardnerella, with significantly higher abundance (1–2 orders of magnitude) compared to non-UTI cohorts. Staphylococcus was also more abundant in UUI.	Specific genera and species, most notably Streptococcus anginosus, showed distinct patterns of prevalence and abundance across these groups. Age and BMI also influence microbiome diversity. The UUI cohort demonstrated the most pronounced differences in microbiome composition compared to other groups.	7
Nardos, 2022 [16]	Case–control study	Women with UUI and healthy controls: 20 women with UUI (cases) and 30 women without UUI (controls).	Permutational multivariate analysis of variance compared microbial community composition; ANCOM identified differentially abundant taxa; and SparCC network analysis examined microbial interactions. The Wilcoxon rank-sum test compared alpha diversity metrics with standard statistical tests for demographics.	PPIUS	Catheterized urine and vaginal samples	UUI: 64.2 years (10.5); Control: 57.9 years (10.4)	PCR using Golay barcoded primers which target the V4 region of 16S rRNA genes	No significant differences in alpha or beta diversity in the bladder microbiome of women with and without UUI were found. The UUI network had fewer genera (93 genera in UUI versus 135 genera in controls) and fewer unique bacterial co-occurrences (624 associations in UUI versus 763 associations in controls). In total, 22 unique genera in UUI and 20 unique genera in the controls were associated with Lactobacillus in their respective networks.	Women with UUI were more likely to have a history of recurrent UTI, which is not surprising given the known overlap in symptoms between UTI and UUI and the frequent misdiagnosis of UTI as a result.	6
Richter, 2022 [17]	Prospective cohort study	Women with MUI enrolled in the ESTEEM trial, who provided catheterized urine and vaginal bacterial samples before mid-urethral sling surgery, with or without perioperative behavioral and pelvic floor therapy (N = 126) (104 provided subjective and 100 provided objective response data at 12 months)	Clinical and demographic differences were assessed using unadjusted general linear models, logistic models, and Wilcoxon rank-sum tests. Generalized linear models evaluated microbiome abundance for predominant genera, while differential abundance analysis and ANOVA with Tukey’s HSD tests examined less abundant genera and incontinence subtypes.	UDI	Catheterized urine and vaginal samples	53.5 years (10.8)	16S rRNA (ribosomal ribonucleic acid) sequencing	Predominant genera Lactobacillus (positive response) and Prevotella (unclear association) were linked to treatment response in unadjusted models, with only Prevotella remaining significant after age adjustment. Several less abundant vaginal bacteria, inversely correlated with Lactobacillus, were associated with poorer treatment response. No significant links were found between urinary microbiome composition and treatment response.	The composition of the vaginal microbiome, particularly the abundance of certain bacterial groups, might be linked to the effectiveness of surgical treatment for MUI.	7
Sun, 2022 [18]	Cross-sectional study	Women with OAB and asymptomatic controls (N = 73) (55 OAB patients and 18 controls)	t-test, Mann–Whitney U-test, and Chi-square test for demographic and clinical characteristics, Spearman correlation for variable relationships, Binary logistic regression is used for predictive analysis, and the LEfSe algorithm is used for bacterial composition analysis.	OABSS and OAB-V8 questionnaires	Catheterized urine	OAB: 43.21 years (4.55), control: 50.5 years (13.34)	Metagenomic next-generation sequencing (mNGS), Kraken2, Bracken 2.0, QIIME2 for diversity indices	Viral infections, including JC virus, were found in 47.3% of OAB patients and linked to worse symptoms (higher OABSS and OAB-V8 scores). Virus-infected patients had increased Staphylococcus warneri, hominis, and epidermidis, reduced bacterial diversity, and lower Bacteroidetes abundance. Correlations showed that viral infections were associated with worse OAB symptoms, age, pelvic surgery history, and the habit of holding urine, which also served as risk factors.	Viral infections in OAB patients are associated with aggravated symptoms and altered urinary bacteriome, indicating the potential role of viruses in the pathophysiology of OAB.	6
Yoshikata, 2022 [19]	Pilot randomized controlled trial	Healthy women (pre- and postmenopausal women) (N = 35) (35 premenopausal and 35 postmenopausal)	T-tests or Mann–Whitney U tests compared continuous variables (e.g., microbiome composition, symptom scores) between groups. Chi-square or Fisher’s exact tests compared categorical variables (e.g., presence/absence of bacteria). Repeated measures ANOVA was used for multiple time points.	OABSS	Vaginal	37.26 years (6.09)	MicroSEQ™ ID 16S rRNA reference database and Greengenes database identified the bacteria down to genus or species level. The alpha diversity scores of the Shannon diversity index were used to describe microbial diversity.	Significant differences in the Lactobacillus composition (72% ± 36.84 versus 10% ± 25.11; *p* < 0.0001) as well as vaginal microbial diversity (0.77 ± 0.73 versus 1.92 ± 1.02; *p* < 0.0001) between the two groups of women. The overactive bladder symptom scores did not change much before and after 4-week treatment in all groups.	The use of Lactobacillus-containing feminine products was associated with improved vaginal ecosystem and urogenital health compared to the control group, especially in those women using feminine gel.	7
Li, 2022 [20]	Cross-sectional study	Female patients with OAB (N = 70)	Baseline differences were assessed using t-tests, Mann–Whitney U, Pearson Chi-square, and Fisher’s exact tests. Bivariate correlations analyzed OABSS scores and bacterial abundance.	OABSS	Catheterized urine samples	Mild symptom group: 37.1 years (11.4); Moderate/severe symptom group: 38.5 years (11.6)	16S rRNA gene sequencing (V3–V4 region), QIIME, Illumina MiSeq; Beta-diversity was assessed using Bray–Curtis, weighted, and unweighted UniFrac distances to compare microbial composition between samples.	Moderate/severe OAB patients had higher bacterial richness and diversity. Notable differences in urinary microbiota composition were noted between groups. Eight bacterial genera correlated with specific OAB sub-symptoms, notably Bosea with Nighttime Frequency (r = 0.472, *p* < 0.001).	The bladder microbiome is closely linked to OAB severity, with higher diversity associated with more severe symptoms. Specific bacterial genera correlate with sub-symptoms.	5
Zhou, 2022 [21]	Quasi-experimental study	Female patients with OAB (N = 64)	Variables were summarized as means ± SD, medians (Q1–Q3), or frequencies (%). Comparisons used t-tests, Mann–Whitney U, Chi-square, Fisher’s tests, and AUC for predictive accuracy.	OABSS	Catheterized urine samples and vaginal sample	Effective group: 37.0 years (1.82); ineffective group: 37.42 years (2.17)	16S rRNA V3–V4 regions of bacterial DNA extracted from urine samples	Fewer Lactobacilli were associated with higher numbers of total leaks (daily total leaks = 2.67 (95% CI: 0.76–4.59; *p* = 0.007) and urgency leaks (daily leaks = 1.75 (95% CI: 0.24–3.27; *p* = 0.02). No significant links were found for stress urinary incontinence episodes or UDI scores.	Urinary microbiome community types, with a higher abundance of Prevotella and Gardnerella and a lower abundance of Lactobacillus, are linked to reduced efficacy of mirabegron in treating OAB.	5.5
Komesu, 2020 [22]	Multicenter cross-sectional study	Female patients diagnosed with MUI and asymptomatic control participants (N = 212); 128 MUI and 84 controls	Dirichlet multinomial mixture (DMM) methods, generalized linear mixed models, and post hoc analyses.	The Urogenital Distress Inventory (UDI) was used to measure incontinence symptom severity in MUI participants	Catheterized urine samples and vaginal sample	53 years (11)	16S rRNA V4–V6 regions of bacterial DNA extracted from urine samples	Women with MUI had distinct urinary microbiota compared to controls, with a lower prevalence of high-lactobacillus types (35% vs. 50%; *p* = 0.03) and a higher prevalence of mixed types (25% vs. 10%; *p* = 0.02). Women under 51 with MUI had more diverse bacterial communities than controls (4.5 vs. 2.1 species; *p* = 0.01). No significant differences were found in women aged 51 and older.	Urinary microbiome community types, particularly those with lower Lactobacillus prevalence and greater microbial diversity, are associated with mixed urinary incontinence in younger women. The urinary microbiome may play a role in the pathophysiology of incontinence and could inform future therapeutic strategies.	8
Price, 2020 [23]	Cross-sectional study	Adult women categorized into Continent Controls, SUI, and UUI (N = 309): Continent Controls (N = 150), SUI (N = 50), and UUI (N = 109)	Pearson, Kendall, Spearman correlation, and Wilcoxon rank-sum test.	UDI score	Catheterized urine	Control: 47 years (14); SUI: 54 years (14); UUI: 61 years (13)	MALDI-TOF mass spectrometry for microbial identification after EQUC protocol	UI cohorts (SUI and UUI) exhibited higher bacterial abundance and richness (Chao1, ACE indices) than controls. Specific species, such as Actinotignum schaalii and Aerococcus urinae, were more abundant in UI cohorts. UDI score correlated with richness indices but not evenness.	Higher microbial diversity is associated with increased UI severity, suggesting a possible link between microbiome composition and incontinence.	6
Thomas-White, 2020 [24]	Quasi-experimental study	Female patients with OAB (N = 62)	Linear mixed effects regression, Spearman’s rho for correlations, and Wilcoxon signed-rank tests.	OABq	Both urine and vaginal (catheterized and voided urine)	Median = 68 (IQR: 62–73)	Expanded Quantitative Urine Culture (EQUC) and relative abundance of taxa evaluated through sequencing	After 12 weeks of estrogen treatment, increased Lactobacillus levels in catheterized urine were linked to improved symptom severity on the OAB-q (*p* = 0.02). No significant changes in diversity or Lactobacillus abundance were found in vaginal swabs, perineal swabs, or voided urine samples.	Urinary microbiome community types, particularly those with increased Lactobacillus levels in the bladder, are associated with modest improvements in urgency urinary incontinence symptoms after estrogen therapy.	8
Gill, 2018 [25]	Blinded prospective cohort	Women with OAB (N = 24) and 22 controls	A GLM with repeated measures analyzed pooled data, and Mann–Whitney tests compared outcomes between patients and controls. Multinomial logistic regression examined microbial species dispersion, while a linear mixed effects model assessed the relationship with pyuria.	ICIQ-LUTS; Whittington Urgency Score; Whittington Pain Questionnaire	Midstream urine	63 years (11)	The genetic method uses 4′-6′-diamidino-2-phenylindole (DAPI) to stain bacterial and host DNA, enabling the identification and visualization of bacteria and mammalian cells. DAPI produces blue fluorescence, detecting intracellular and extracellular bacteria without permeabilization. This method enhances bacterial identification localization and distinguishes host and microbial DNA for improved analysis of microbial interactions.	Consistent differences in bacterial ecology, inflammation, and infection markers between OAB patients and controls were observed. OAB patients differ consistently from controls, demonstrating differences in bacterial ecology (t = −4.57; *p* 0.0001), in the microscopic pyuria count (t −6.37; *p* 0.0001), and presence of infected urothelial cells (t −4.21; *p* 0.0001).	While routine urine culture failed to differentiate the groups, enhanced sediment culture, microscopy, and symptom questionnaires revealed clear differences, suggesting a potential role for altered bladder microbiome in OAB pathophysiology. The study challenges the reliance on routine culture for diagnosing UTI in OAB patients.	5
Komesu, 2018 [26]	Multi-site cross-sectional study	Women with and without MUI (N = 207) MUI (N = 123) Control (N= 84)	Descriptive statistics, frequencies, and percentages summarize data while clustering analysis uses Euclidean distance. Strain-level comparisons are mentioned, but the statistical method is unspecified.	UDI	Catheterized urine	53.0 years (10.8)	Variable regions 4–6 of the 16S rRNA gene were amplified by PCR using primers 515F and 1114R with the addition of Illumina^®^ Nextera linker sequences.	Lactobacillus predominance showed no significant difference between MUI (36.6%) and controls (42.9%) (*p* = 0.364). Six DMM community types were identified, with significant distribution differences between MUI and controls (*p* = 0.032) and associations with age, smoking, BMI, and Hispanic ethnicity. Alpha and beta diversity did not differ between groups, but alpha diversity varied by DMM community type.	Lactobacillus predominance was similar between MUI and controls, but microbiome composition, especially in younger women, was linked to MUI. “Mixed” or “Moderate-lactobacillus” types increased MUI odds, while BMI remained a consistent risk factor across age groups.	6
Curtiss, 2017 [27]	Cross-sectional study	Women with OAB vs. healthy controls: 60 OAB and 35 controls	Fisher’s exact test and unpaired Student’s t-test	Mild, moderate, and severe (severity as per ICIQ and PGI-S)	Midstream urine	OAB: 59.2 years (range: 14–87); Controls: 53.7 years (range: 41–83)	PCR, 16S rRNA gene sequencing	Differences in microbiome composition between OAB patients and controls were statistically significant. The bladder microbiome in women with OAB showed significantly different bacterial prevalence compared to controls. Lactobacillus was less common in OAB patients (20% vs. 43%), and Proteus was more prevalent (23% vs. 3%). A total of 66 genera were identified.	Lactobacillus may be protective against OAB, while Proteus is a factor in some OAB cases.	7
Thomas-White, 2017 [28]	Cross-sectional study	Female patients with uncomplicated SUI (N= 197)	Generalized Estimating Equations (GEEs) to assess the association between urinary microbiota diversity and demographic or symptom measures.	MESA stress and urge index score	Urine (voided and catheterized)	51 years (9.7)	16S rRNA V4 region of bacterial DNA extracted from urine samples	Increased microbial evenness was linked to higher MESA urge index scores (0.03 units; *p* = 0.04), higher BMI (0.1 units; *p* = 0.02), and post-menopausal hormone-negative status (0.23 units; *p* = 0.004). No significant links were found with SUI symptoms.	Urinary microbiome community types, particularly those with increased microbial diversity and evenness, are associated with urgency urinary incontinence symptoms but not stress urinary incontinence symptoms.	5
Wu, 2017 [29]	Cross-sectional study	Female patients with OAB; OAB: 30; Controls: 25	Descriptive statistics, chi-square tests, and multivariate regression analysis.	OAB-Q and the 3-day bladder diary	Urine via transurethral catheterization and vaginal samples	OAB: median: 27.5 years (IQR: 26.0, 35.3); Controls: median: 26.0 (IQR: 23.0, 47.5)	16S rRNA V3–V4 regions of bacterial DNA extracted from urine samples	Fewer Lactobacilli were linked to higher OAB-Q scores (*p* = 0.03) and more urgency incontinence episodes (2.56 daily leaks; 95% CI, 0.68–4.44; *p* = 0.01). No significant links were found for total leaks or other incontinence severity measures.	Urinary microbiome community types, particularly those with a lower abundance of Lactobacillus, are associated with increased severity of overactive bladder symptoms and more frequent urgency incontinence episodes.	5.5
Karstens, 2016 [30]	Case–control study	Women with UUI, and women with normal bladder function; (N = 20); 10 UUI cases and 10 controls	Baseline differences were assessed using t-tests and Fisher’s exact test. Microbiome analysis included diversity, relative abundances and clustering. Differential OTUs between UUI and controls were identified using DESeq2 (FDR-adjusted *p* < 0.1).	The 3-day bladder diary (frequency of urination, leakage episodes, fluid intake, and urgency severity scale), ICIQ, UDI, PFIQ, OABQ, and USS	Urine samples using a transurethral catheter	Controls: 58 years (9), UUI: 57 years (8)	DNA extraction, sequencing of 16S rRNA V4 region, PCR amplification with GoTaq HotStart Polymerase, Illumina MiSeq sequencing, QIIME, FASTQ-join, RT-qPCR	Microbiome analysis revealed no significant differences in bacterial DNA concentration, but there were differences in bacterial composition (e.g., increased Proteobacteria in UUI). Decreased microbial diversity in UUI correlated with symptom severity (e.g., UDI).	The urinary microbiome may play a role in the pathophysiology of UUI. Decreased diversity may be linked to disease severity and may serve as a potential therapeutic target.	8
Thomas-White, 2016 [31]	Open-label clinical trial	Female patients with UUI and a control group of women without UUI (N= 134); 74 cases and 60 controls at baseline	Continuous data: means ± SD or medians (IQR); categorical: frequencies. Response analysis required 12-week data. Fisher’s exact, Kruskal–Wallis, Wilcoxon, and Kappa tests were used.	PGSC questionnaire and OAB-Q	Urine via transurethral catheter	UUI participants: 61.5 years (11.5); Controls: 49 years (14.7)	16S rRNA V4 region of bacterial DNA extracted from urine samples and expanded quantitative urine culture	Women with UUI had more diverse urinary microbiota than controls, with fewer Lactobacilli and higher microbial diversity.	Urinary microbiome community types, particularly those with fewer Lactobacillus and higher diversity, are associated with urgency urinary incontinence severity and reduced treatment response to solifenacin.	9
Pearce, 2015 [32]	Multi-site randomized trial	Women with UUI (N = 182)	Baseline differences and outcomes were analyzed for sequence-positive/-negative individuals and by urotype using χ2 tests and general linear models. The Wilcoxon rank-sum test compared to the top 10 taxa abundances.	Episodes of UUI, UDI scores	Catheterized urine samples	55.8 years (12.2) for sequence-positive; 61.3 years (9.0) for sequence -negative	Sequencing of 16S rRNA gene (V4 region) using Illumina MiSeq, DNA extraction from urine, PCR amplification, sequencing, mothur and METAGENassist.	Lactobacillus was dominant in most samples. The higher bacterial richness and diversity were associated with moderate/severe UUI.	Microbiota may influence UUI severity and treatment response. Lactobacillus-dominant microbiota associated with better treatment outcomes.	7

## Data Availability

No new data were created for this study.

## Abbreviations

The following abbreviations are used in this manuscript:

AUC	Area Under the Curve
BMI	Body Mass Index
ICIQ	International Consultation on Incontinence Questionnaire
LUTS	Lower Urinary Tract Symptoms
MESA	Medical, Epidemiologic, and Social Aspects of Aging
MUI	Mixed Urinary Incontinence
NOS	Newcastle–Ottawa Scale
OAB	Overactive Bladder
OAB DO	Overactive Bladder Detrusor Overactivity
OABQ	Overactive Bladder Questionnaire
OABSS	Overactive Bladder Symptom Score
OTU	Operational Taxonomic Unit
PFIQ	Pelvic Floor Impact Questionnaire
PGSC	Patient Global Symptom Control
PPIUS	Patient Perception of Intensity Urgency Scale
PRISMA	Preferred Reporting Items for Systematic Review and Meta-analyses
SDI	Shannon Diversity Index
SUI	Stress Urinary Incontinence
UDI	Urogenital Distress Inventory
USS	Urgency Severity Scale
UUI	Urgency Urinary Incontinence
UDI	Urogenital Distress Inventory
